# miR-26b enhances the sensitivity of hepatocellular carcinoma to Doxorubicin via USP9X-dependent degradation of p53 and regulation of autophagy

**DOI:** 10.7150/ijbs.52517

**Published:** 2021-02-08

**Authors:** Enjiang Chen, Enliang Li, Hao Liu, Yue Zhou, Liang Wen, Jianxin Wang, Yi Wang, Longyun Ye, Tingbo Liang

**Affiliations:** 1 The Second Affiliated Hospital of Zhejiang University, School of Medicine, Hangzhou, China.; 2 Zhejiang Provincial Key Laboratory of Pancreatic Disease, Hangzhou, China.; 3 Department of Hepatobiliary and Pancreatic Surgery, The First Affiliated Hospital, Zhejiang University School of Medicine, Hangzhou, China.; 4 Department of Medical Oncology, Tongde hospital of Zhejiang Province, Hangzhou, Zhejiang, 310012, China.; 5 Department of Pancreatic Surgery, Fudan University Shanghai Cancer Center, Shanghai 200032, China.; 6 Zhejiang Provincial Innovation Center for the Study of Pancreatic Disease, Hangzhou, China.; 7 Zhejiang Clinical Research Center of Hepatobiliary and Pancreatic Diseases, Hangzhou, China.

**Keywords:** microRNA-26b, hepatocellular carcinoma, Doxorubicin, USP9X, p53.

## Abstract

Multi-drug resistance is a major challenge to hepatocellular carcinoma (HCC) treatment, and the over-expression or deletion of microRNA (miRNA) expression is closely related to the drug-resistant properties of various cell lines. However, the underlying molecular mechanisms remain unclear. CCK-8, EdU, flow cytometry, and transmission electron microscopy were performed to determine cell viability, proliferation, apoptosis, autophagic flow, and nanoparticle characterization, respectively. In this study, the results showed that the expression of miR-26b was downregulated following doxorubicin treatment in human HCC tissues. An miR-26b mimic enhanced HCC cell doxorubicin sensitivity, except in the absence of p53 in Hep3B cells. Delivery of the proteasome inhibitor, MG132, reversed the inhibitory effect of miR-26b on the level of p53 following doxorubicin treatment. Tenovin-1 (an MDM2 inhibitor) protected p53 from ubiquitination-mediated degradation only in HepG2 cells with wild type p53. Tenovin-1 pretreatment enhanced HCC cell resistance to doxorubicin when transfected with an miR-26b mimic. Moreover, the miR-26b mimic inhibited doxorubicin-induced autophagy and the autophagy inducer, rapamycin, eliminated the differences in the drug sensitivity effect of miR-26b. *In vivo*, treatment with sp94dr/miR-26b mimic nanoparticles plus doxorubicin inhibited tumor growth. Our current data indicate that miR-26b enhances HCC cell sensitivity to doxorubicin through diminishing USP9X-mediated p53 de-ubiquitination caused by DNA damaging drugs and autophagy regulation. This miRNA-mediated pathway that modulates HCC will help develop novel therapeutic strategies.

## Introduction

Hepatocellular carcinoma (HCC) has a high-incidence and aggressive types of cancer, with increasing global incidence, ranking second in mortality worldwide 1, 2; however, several treatment methods can cure patients suffering from HCC, including liver transplantation, radiotherapy, surgery, and interventional therapy. In addition, chemotherapy is often used before or after the above-mentioned treatment strategies. Due to the ineffective chemotherapy or multidrug resistance (MDR) procedures, patients with advanced HCC have a poor prognosis or clinical outcomes 3. Therefore, it is necessary to identify a new molecular mechanism underlying the drug resistance and develop effective treatment strategies for HCC.

MicroRNAs (miRNAs) are a type of evolutionarily conserved non-coding small RNA with the function of regulating gene expression at the stage of translation, and has been considered to be abnormally expressed in several human cancers 4, 5. Accumulating research indicates that some miRNAs display aberrant expression in various cancer cells (e.g., HCC, breast cancer, and human non-small lung cancer [NSCLC]) 6-8. In several cancers, miRNAs act as tumor suppressors or oncogenes related to the multiple pathways and cell functionality involved in development and progression of cancer (e.g., cell cycle, proliferation, and apoptosis) 9, 10. It has been demonstrated that miRNA-26b (miR-26b) is involved in a variety of malignant cancer progressions as tumor oncogenes or suppressors (e.g., lung, HCC, melanoma, ovarian, and colorectal cancer) 11-15. Importantly, the aberrant expression of miR-26b is significantly associated with cell proliferation, metabolism, and apoptosis 16. These results are largely due to their effect of the activation of multiple related downstream target genes on angiogenesis, the process of cell-cycle development and anti-apoptosis 17, 18.

Ubiquitination has been found to be a key regulatory mechanism in multiple biological processes and controls almost all aspects of protein function through the reversible post-translational modification of cellular proteins by the activity of ubiquitylating and deubiquitylating enzymes (DUBs)^19^, ^20^. Ubiquitin-specific proteases (USPs) represent the largest DUB group of ubiquitinating enzymes, which function to specifically depolymerize ubiquitin from ubiquitinated substrates and play an important role in the ubiquitin system. Ubiquitin-specific protease-9 (USP9X) acts as a member of the USP family to regulate cell sensitivity to chemo and radiotherapies. Moreover, USP9X was found to be overexpressed in different types of lymphoma, such as diffuse large B-cell and follicular lymphoma, as well as multiple myeloma 21. WP1130 acts as a partially selective DUB inhibitor, and is considered to be a potential chemosensitizer due to its inhibitory activity on USP9X de-ubiquitination 22, 23. In addition, WP1130 can promote the down-regulation of myeloid cell leukemia 1 protein (Mcl-1) and increase the sensitivity of tumor cells to chemotherapy 24. WP1130 also increases sensitivity to doxorubicin via degrading p53 by a USP9X-dependent mechanism in HCC cells 22. Autophagy is a highly complex process, which plays a dual role in the processes of tumor treatment, regulation of cell survival, differentiation, apoptosis, and a series of other biological processes 25, 26. Moreover, miRNA may participate in the resistance of various cancers to therapy 27, 28. It has been previously reported that miR-26a/b enhances apoptosis and sensitivity in HCC via inhibiting ULK1 29. Xinming et al. demonstrated that the overexpression of miR-30 enhances cisplatin sensitivity through the inhibition of autophagy 30.

This study evaluated the miR-26b-induced sensitivity of HCC cells to doxorubicin. Furthermore, we confirmed the specific mechanism of miR-26b on HCC cells through USP9X down-regulates p53 upregulation caused by DNA damaging drugs and the regulation of autophagy.

## Materials and Methods

### Cell culture and the Source of HCC tissue

Human HCC cells (HepG2 (Wild-type p53), Hep3B (p53 deletion) SNU387 and SNU449 (p53 mutation) were supplemented from ATCC. All of the HCC cells were maintained in different medium containing 10% FBS(GIBCO) and 1% penicillin/streptomycin (Sigma), such as SNU449 and SNU387 (RPMI1640 medium), HepG2 (DMEM), Hep3B (MEM). The cells were cultured in a humidified environment containing 5% CO2 at 37°C. The adjacent tissues and HCC tissues were obtained from The Second Affiliated Hospital of Zhejiang University, School of Medicine.

### Transient transfection with an miR-26b mimic and inhibitor

According to the manufacturer's instructions, Lipofectamine 2000 (Invitrogen,) was used to transfect the HCC cells. For the SP-94dr/miR-26b nanoparticle infection, miR-26b mimics and SP-94dr were mixed and allowed to rest for 15 min, after which they were added to the plates. RiboBio (Ribobo Co., Ltd.) and Santa Cruz Biotechnology (Santa Cruz, CA, USA) provided the related transient transfection products (miR-26b mimic, miR-26b inhibitor, and negative control: RiboBio; USP9X siRNA , negative siRNA : Santa Cruz).

### Cell viability assay

Cell Counting Kit-8 (CCK-8; Dojindo) was used to determine the related cell viability according to product description. Briefly, HCC cells (3 × 103 cells/well) were seeded into 96-well plates and cultured for 24 h. The culture medium was replaced by 10% FBS-medium containing the indicated doxorubicin concentration. After a further incubation for 48 h, 10 μL of CCK-8 solution was added, the cells were incubated for an additional 3 h, and then absorbance at 450 nm was measured using an MRX II microplate reader (Dynex Technologies, Chantilly, USA). Relative cell viability was calculated as a percentage of the untreated controls.

### Western Blot analysis

The HCC cells transfected with the miR-26b mimic, or USP9X siRNA were lysed with RIPA lysis buffer (Beyotime, Shanghai, China), centrifuged at 12,000 rpm for 10 min at 4°C, and the supernatants were collected. After collecting the protein samples, the protein concentration of each sample was determined to ensure that the amount of each protein sample was consistent. Then protein was quantified using a BCA protein kit. A 10% SDS-PAGE gel was prepared and the same amount of protein was loaded in to each well. The separated proteins were subsequently transferred onto a PVDF membrane. The membrane was slowly agitated on a shaker, blocked in 5% TBST at room temperature for 60 min, washed three times, and incubated with a 1:1,000 diluted primary antibody (anti-USP9X, anti-p53; Abcam) in TBST overnight at 4°C on a side-swing shaker. The membrane was washed three times for 5 - 10 min with slow shaking on the side-swing shaker before incubating with the corresponding secondary antibody (Abcam; 1:2000) at room temperature or 4°C with slow shaking for 1 h on a side swinging shaker. Finally, the detection of the proteins was performed using an ECL color kit. A gray value quantification analysis of the protein bands was performed using ImageJ software (National Institutes of Health, Bethesda, MD, USA).

### Quantitative real-time reverse transcription-PCR (RT-PCR)

Total RNA was extracted using TRIzol reagent (Invitrogen) according to the manufacturer's instructions. Reverse transcription was performed to obtain the first strand of cDNA using a PrimeScript® RT reagent kit (Takara). The relative level of miR-26b was normalized to U6. All qRT-PCR reactions were performed using SYBR Green Master Mixes (Thermo Fisher). The results were analyzed using the 2^-ΔΔCt^ method. The primer sequences are listed as follows: p53: Forward 5'-TCAGCATCTTATCCGAGTGGAA-3'; Reverse 5'-TGTAGTGGATGGTGGTACAGTCA-3'. USP9X: Forward 5'-CAATGGATAGATCGCTTTATA-3'; Reverse 5'-CTTCTTGCCATGGCCTTAAAT-3'. hsa-miR-26b mimics: Forward 5'-UUCAAGUAAUUCAGGAUAGGU-3'; Reverse 5'-CUAUCCUGAAUUACUUGAAUU -3'. hsa-miR-26b inhibitor: 5'-ACCUAUCCUGAAUUACUUGAA-3'.

### Cell proliferation analysis

Proliferation of HCC cells was determined using an EdU staining proliferation kit according to manufacturer's (Abcam) protocols. The HCC cells (3 × 103 cells/well) transfected with or without miR-26b mimics were seeded into 96-well plates and cultured for 24 h. Later, the cells were treated with or without doxorubicin for 24 h and incubated with 20 μM EdU for 3 h. DNA (blue) was stained with Hoechst 33342. Green cells indicated EdU/Hoechst-positive cells.

### Flow cytometry

The apoptosis rate of the HCC cells was determined using an Annexin V-FITC Apoptosis Detection Kit (Abcam). Briefly, HCC cells (treated as above) were harvested by trypsinization, rinsed with ice-cold PBS, and centrifuged to remove the supernatant. The cells were resuspended in 100 μL 1× binding buffer and incubated with Annexin V-FITC for 15 min in the dark at room temperature. Finally, flow cytometry was used to determine the number of apoptotic cells based on the B2 and B4 quadrants.

### Transmission electron microscopy

Autophagosome formation of was examined using transmission electron microscopy (TEM). The harvested cells were fixed with 2.5% glutaraldehyde at 4°C overnight, and post-fixed in 1% buffered osmium tetroxide for 2 h. After dehydration in series of ethanol, the samples were embedded in an epoxy resin, followed by staining with uranyl acetate and lead citrate. The stained sections were examined under a transmission electron microscope (H800; Hitachi).

### Luciferase Reporter Assay

The amplified of the wild type USP9X-3'UTR or mutant USP9X-3'UTR fragment was cloned into the pGL3 vector containing the firefly luciferase reporter gene (Promega, Madison, WI, USA). For the luciferase reporter assays, HEK293 cells were plated in 96-well plates and transiently co-transfected with 200 ng firefly luciferase construct, 4 ng pRL-TK Renilla luciferase plasmid, and 50 nM miR-26b-mimic according to the manufacturer's protocol supplied with a luciferase reporter assay kit (Promega Madison, WI). After 48 h transfection, the relative Renilla luciferase activity (firefly luciferase/Renilla luciferase) was measured with a dual luciferase reporter assay (Promega, USA).

### Nanoparticle characterization

SP-94dr/miR-26b nanoparticles were analyzed for hydrodynamic diameters and zeta-potential using a laser particle analyzer at 25°C. TEM was performed at an accelerating voltage of 80 kV with a Philips TECNAL-10 (Eindhoven).

### MiRNA stability test

A total of 20 μM miR-26b and SP-94dr (SP-94dr: miR-26b) was mixed at a molar ratio of 20:1 and incubated at room temperature for 10 min, 1 μL RNase A (diluted to 0.001 μg/μL, Takara) was added and incubated for the indicated times at room temperature and separated on a 2% agarose gel.

### Nude mouse xenograft model

BALB/c nu/nu mice (4-5 weeks old) were obtained from GemPharmatech Co., Ltd (Nanjing, China). Patient-derived tumor xenografts (PDTX) were used to establish the tumor model. PDTX maintains the morphological, structural, and molecular characteristics of the tumor cells. Furthermore, PDTX uses a transplant method that is closer to the tumor microenvironment. In our research group, the rate of successful PDTX tumor inoculation was about 10%, 20 case HCC tumor tissue can be successfully modeled in 2 cases. Tumor fragments of 1 mm3 were cut from resected tumors and inoculated into the mice. After the tumor had formed, the tumor tissue was obtained, sliced, and repeatedly inoculated into 32 BALB/c nude mice, at last we used the tumor into BALA/c nude mice to do the further research. When the diameter of tumors reached a size of 0.5 cm, and the tumor volume reached about 50 mm^3^ -100 mm^3^, the mice were randomly divided into four groups: 1) Control (saline); 2) Doxorubicin (2 mg/kg); 3) miR-26b mimic (sp-94dr) (miR-26b mimic: 10 μg, sp-94dr: 200 μg, the injection dose of the mixture was 100 μL each); and 4) miR-26b mimic (sp-94dr) + Doxorubicin (n = 6 per group). In this experiment, the peptide nanoparticles formed by the mixture of mir-26b mimics and sp-94dr peptides were injected into the tail vein. The control (normal saline), Doxorubicin, miR-26b mimic, or miR-26b mimic plus Doxorubicin groups were administered multi-point intratumoral injections every other day for two weeks. The tumor volume was recorded every two days (Tumor volume (mm^3^) = length × width^2^/2). The mice were sacrificed on Day 15 after treatment and the tumors were weighed.

### Immunocytochemistry

Immunohistochemical staining was performed to determine the level of Ki-67 expression. The paraffin sections were deparaffinized in water, blocked with 5% - 10% normal goat serum (diluted in PBS) for 10 min at room temperature, and incubated with an anti-Ki-67 antibody (1:500, Abcam) at 4°C overnight. The samples were washed with PBS three times, incubated with horseradish peroxidase (HRP) for 30 min at 37°C, washed again, and diaminobenzidine (DAB) was added and developed for 10 min. Finally, hematoxylin was used as a counterstain, the sections were dehydrated, sealed in film, and observed under light microscopy (Olympus).

### TUNEL analysis

Apoptosis in paraffin-embedded mouse tissue sections (5-mm) was determined using an *in situ* cell death detection kit (Roche). Briefly, the cells were fixed in 4% paraformaldehyde for 24 h. After conventional dehydration, wax infiltration, and paraffin embedding, the cells were subsequently incubated with a mixture of a fluorescent labeling solution of 450 μL dUTP and 50 μL TdT enzyme at 37°C for 1 h. Next, 50 μL converter-POD was added to the specimen, a cover glass or parafilm was added and reacted in a dark humidified box at 37°C for 30 min. The samples were subsequently treated with diaminobenzidine for 10 min at 15°C - 25°C, counterstained with hematoxylin (to identify the cell nuclei), dehydrated in a gradient series, vitrified with dimethylbenzene, and finally, mounted with neutral balsam. The prepared samples were evaluated under an optical microscope.

### Statistical analysis

The experimental data are presented as the mean ± SD. Comparisons between two groups or multiple groups were analyzed using a two-tailed Student's *t*-test and a one-way analysis of variance (ANOVA) followed by Tukey's post hoc test, respectively. A value of P less than 0.05 was identified as a statistically significant difference. Statistical analysis was performed using GraphPad Prism 8. All of the experiments were repeated at least three times.

## Results

### Treatment with miR-26b enhances HCC cell sensitivity to doxorubicin

**Figure 1:**
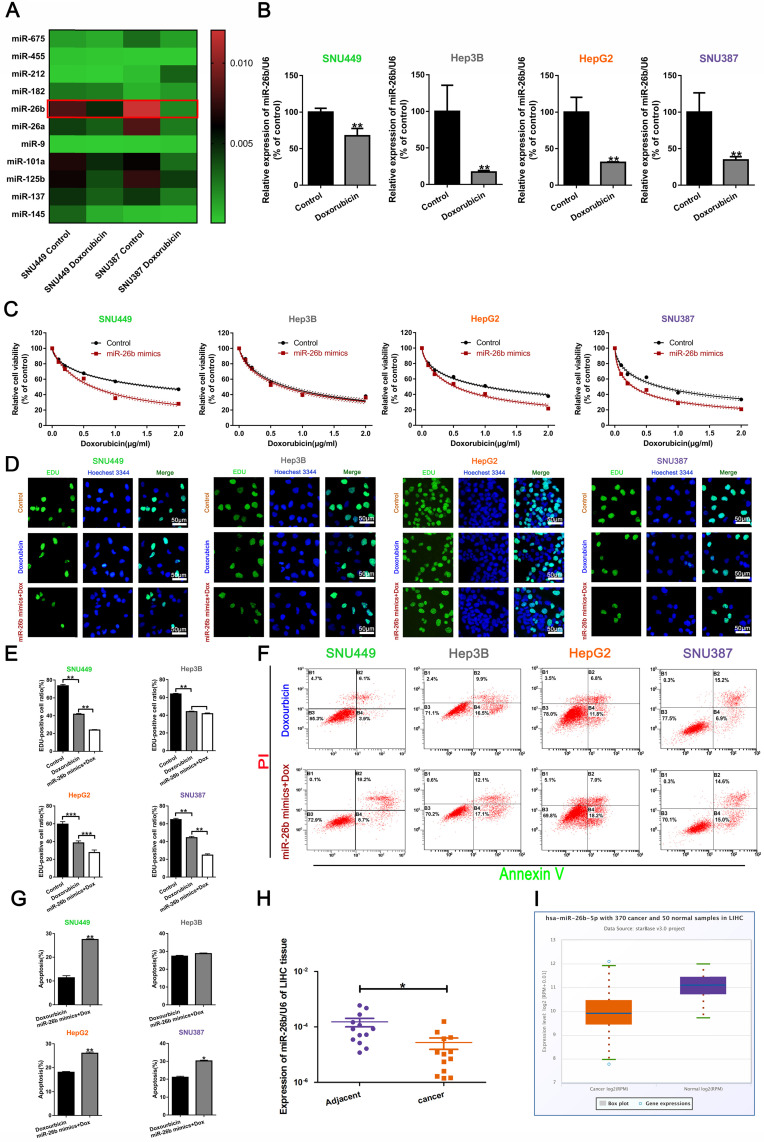
** miR-26b enhances HCC cell sensitivity to doxorubicin. A.** QRT-PCR analysis of the changes in miRNA after treatment with doxorubicin in SNU449 and SNU387 cells. **B.** The level of miR-26b was determined following treatment with or without Doxorubicin by qRT-PCR in HCC cells. **P < 0.01.** C.** A CCK-8 assay analysis showed that treatment with an miR-26b mimic can enhance the sensitivity of HCC cells to doxorubicin, with the exception of Hep3B cells.** D and E.** An EdU incorporation assay of cellular proliferation in different treatment groups. **P < 0.01.** F and G.** The apoptosis ratio was determined by flow cytometry. *P < 0.05; **P < 0.01.** H.** QRT-PCR used to determine miR-26b expression in adjacent cancer and adjacent tissues.** I.** We used StarBase v 3.0 project to analyze the level of miR-26b in normal and cancer tissues.

QRT-PCR was used to analyze changes in miRNA before and after doxorubicin treatment in SNU449 and SNU387 cells. MiR-26b expression was the lowest in the cells treated with doxorubicin (**Figure 1A**). To reveal the role of miR-26b on HCC cell doxorubicin sensitivity, qRT-PCR was used to determine the level of miR-26b in HCC cells, and we found that the level of miR-26b was down-regulated after treatment with doxorubicin** (Figure 1B).** Treatment with the miR-26b mimic could enhance the sensitivity of SNU449, HepG2, and SNU387 cells to doxorubicin, whereas the miR-26b mimic had no effect on the sensitivity to doxorubicin in Hep3B cells **(Figure 1C).**

The IC50 values are listed in **Table 1.** EdU incorporation assay and the flow cytometry analysis indicated that the miR-26b mimic combined with doxorubicin could reduce the cellular proliferation rate and increase the number of apoptotic cells in HCC cells, except Hep3B cells **(Figure 1D-G)**. Almost immediately, we found that miR-26b was expressed to a greater extent in the adjacent tissues **(Figure 1H).** StarBase v.3 was used to analyze miR-26b expression in liver hepatocellular carcinoma (LIHC) 31, 32, showing that miR-26b was expressed to a greater extent in the normal tissues compared to that in cancer tissues **(Figure 1I).**

### The relationship between p53 and doxorubicin sensitivity in HCC cells

**Figure 2:**
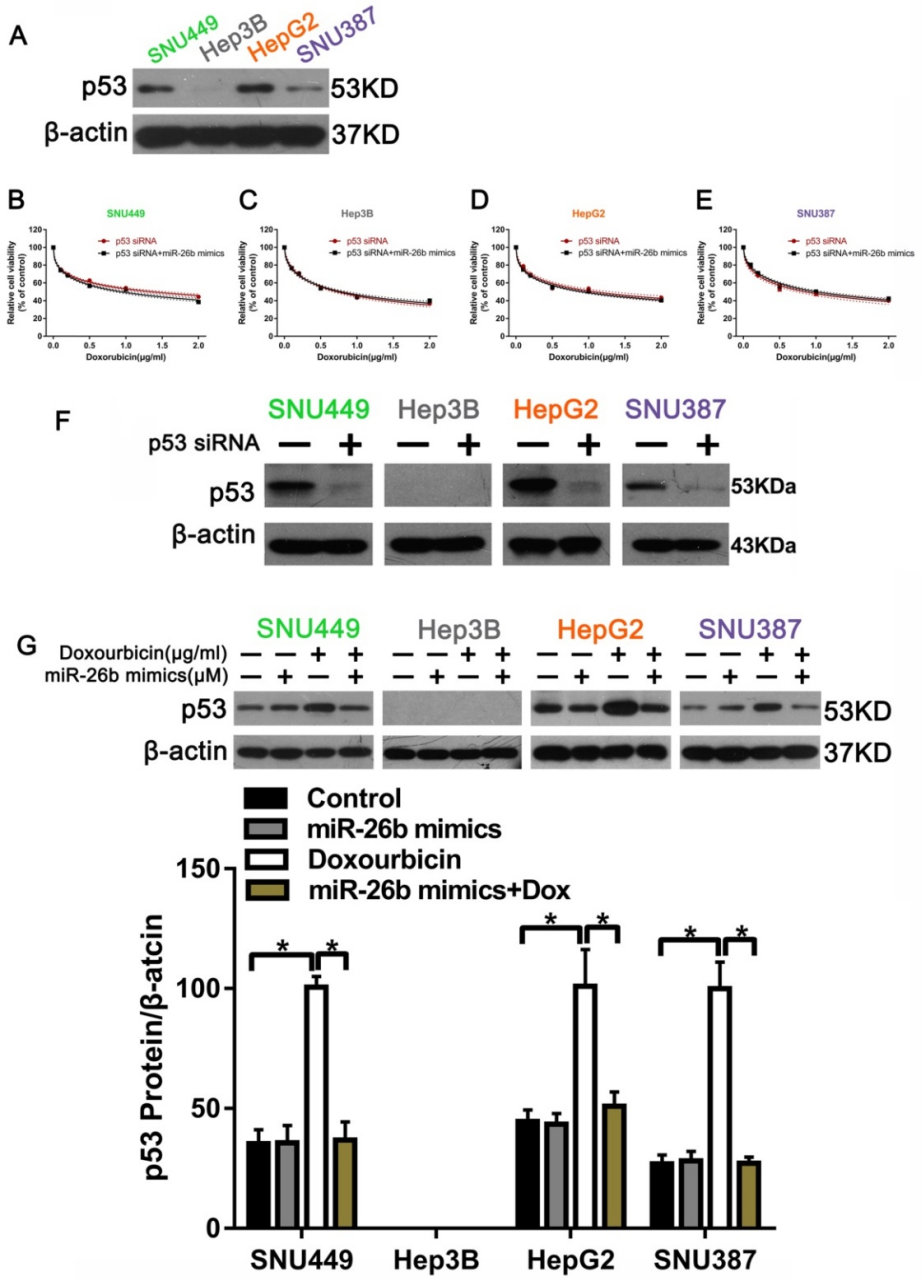
** p53 was related to the sensitivity of HCC cells to doxorubicin. A.** P53 protein expression was detected by Western blot. **B - E.** The cell viability was examined in doxorubicin, or miR-26b mimic plus doxorubicin treated cells following transfection with p53 siRNA.

Our previous research demonstrated that WP1130 increases doxorubicin sensitivity in HCC cells through USP9X-dependent p53 degradation 22. We assumed that whether or not miR-26b had an effect on doxorubicin treatment was due to p53 degradation. To test this theory, we first examined p53 expression in different HCC cells by Western blot. The results showed that the HCC cells expressed p53, except Hep3B cells** (Figure 2A)**. We also transfected p53 siRNA into HCC cells and observed the level of cell viability between doxorubicin treatment alone and the miR-26b mimic plus doxorubicin treatment, revealing that the effect of miR-26b mimic enhanced doxorubicin sensitivity in HCC cells was inhibited following transfection with p53 siRNA **(Figure 2B-E)**. The Western blot confirmed the detection interference efficiency of p53** (Figure 2F).** As shown in **Figure 2G**, as compared to doxorubicin treatment alone, p53 expression was down-regulated after transfection with the miR-26b mimic and interaction with doxorubicin.

### miR-26b enhances HCC cell sensitivity to doxorubicin via USP9X-dependent p53 degradation

**Figure 3:**
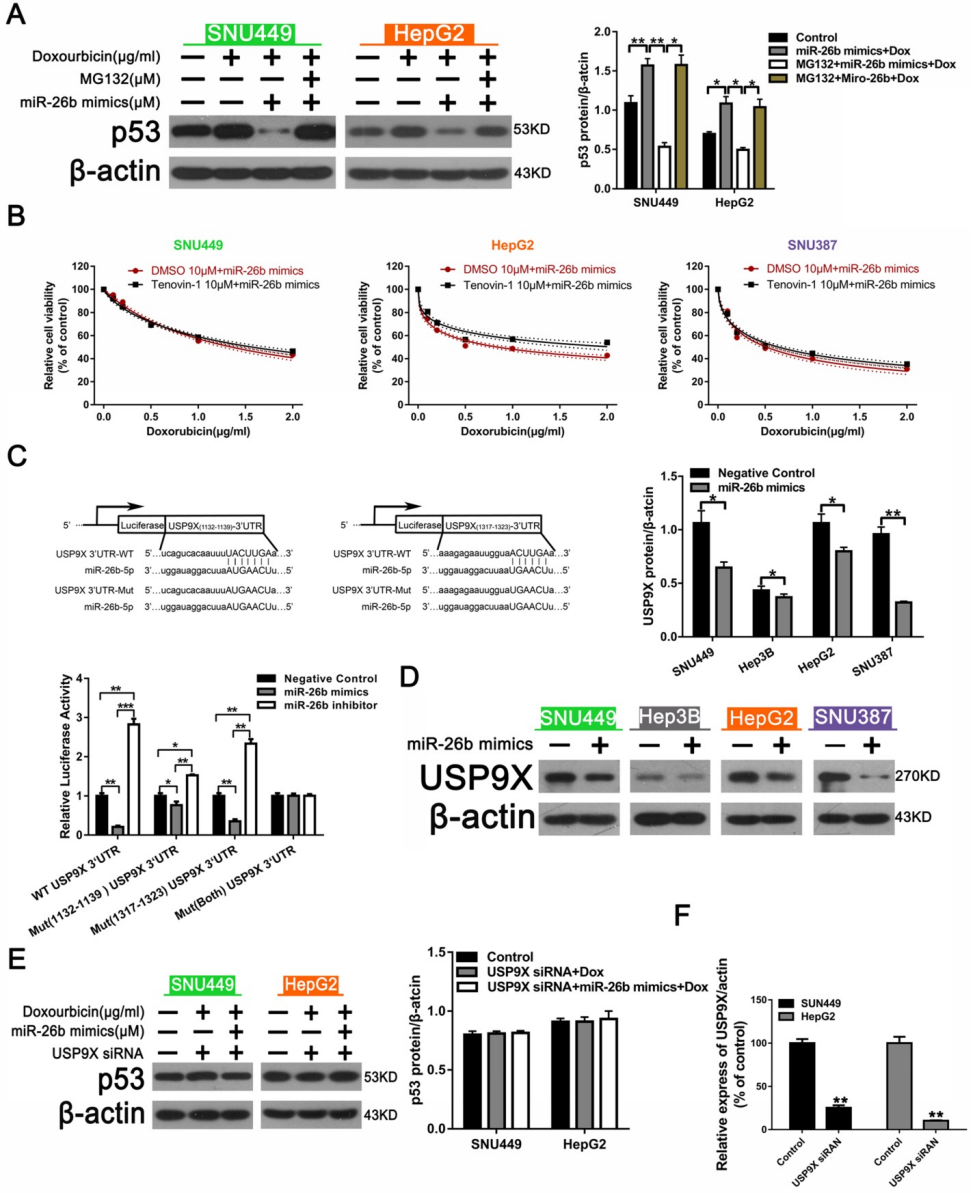
** miR-26b enhances HCC cell sensitivity to doxorubicin via USP9X-dependent p53 degradation. A.** Western blot indicating the expression of p53 after treated with doxorubicin, doxorubicin plus miR-26 mimic, or MG132. **P < 0.01, ***P < 0.001. **B.** Tenovin-1 combined with an miR-26b mimic could enhance doxorubicin sensitivity in HepG2 cells, except other HCC cells. **C.** The predicted miR-26b binding site in the USP9X 3′ UTR and dual fluorescence reporter gene experiments verify that miR-26b binds to the promoter region of USP9X. *P < 0.05, **P < 0.01, ***P < 0.001. **D.** The expression of USP9X was up-regulated following treatment with the miR-26b mimic. *P < 0.05, **P < 0.01. **E.** P53 protein expression was determined by Western blot. F. The expression of UXP9X was determined by Western Blot.

We next further explored the mechanism by which miR-26b regulates doxorubicin-induced p53 expression. As shown as in** Figure 3A**, p53 was down-regulated in the miR-26b mimic + doxorubicin group, whereas p53 was re-covered following treatment with MG132 + miR-26b MIMIC + doxorubicin group, suggesting that MG132 could block the activity of miR-26b, and miR-26b may play a role in promoting p53 ubiquitination degradation. In addition, the CCK-8 analysis showed that in all of the HCC cells, except in the HepG2 cells treated with Tenovin-1 (a wild type p53 activator) for 2 h, miR-26b could reduce the sensitivity to doxorubicin in HepG2 cells **(Figure 3B)**. We further explored the mechanism by which miR-26b regulates doxorubicin-induced p53. Targetscan software predicted that miR-26b could bind to the USP9X promoter region and dual luciferase reporter gene experiments verified that miR-26b overexpression decreased the luciferase activity of the constructs containing the wild type USP9X 3′UTRs, Mut (1132-1139), or (1317-1323) USP9X 3′UTRs, whereas Mut(both) USP9X 3′UTRs remained unchanged** (Figure 3C)**. We assumed that miR-26b could regulate p53 degradation by USP9X. A Western blot revealed that USP9X expression was down-regulated after transfection with the miR-26b mimic **(Figure 3D).** The level of p53 expression between the doxorubicin + USP9X siRNA group and doxorubicin + USP9X siRNA + miR-26b mimic were not significantly different** (Figure 3E)**. Furthermore, the interference efficiency of USP9X was determined by Western Blot (Figure 3F).

### miR-26b inhibits doxorubicin-induced autophagy

**Figure 4:**
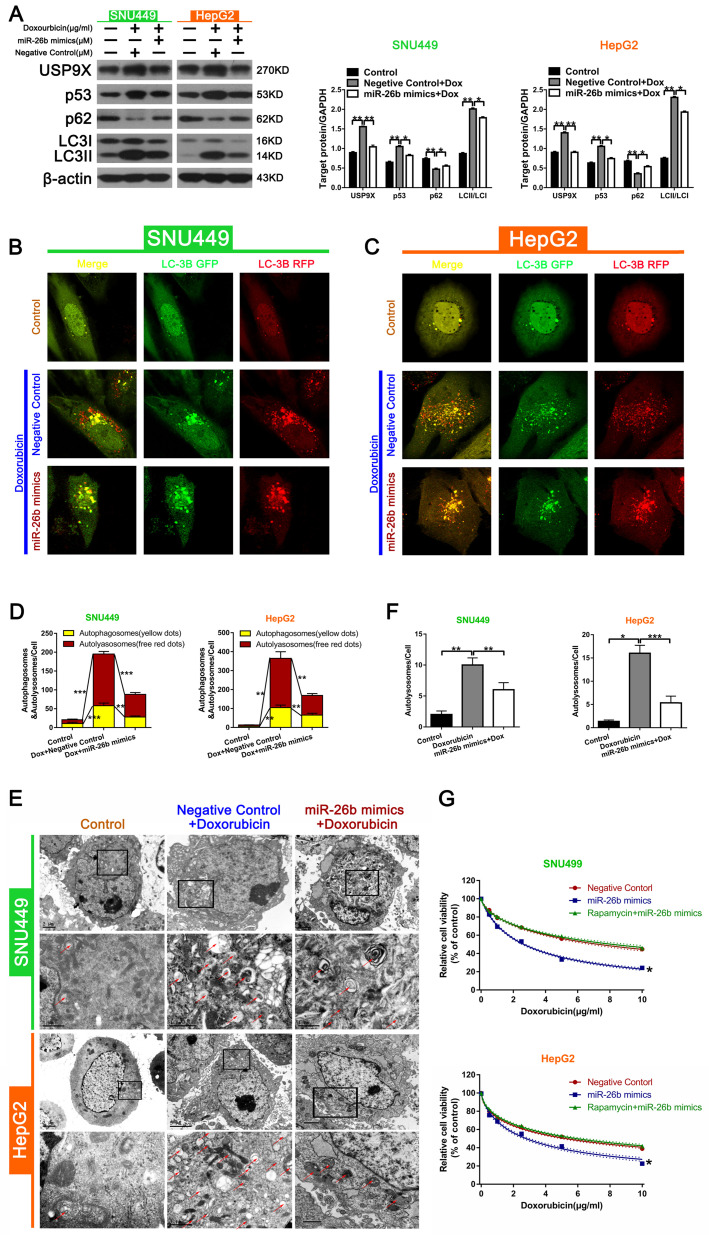
** miR-26b enhances doxorubicin sensitivity via autophagy. A.** The expression of proteins in HCC cells in different treatment groups detected by Western blot. *P < 0.05, **P < 0.01.**B-D.** Confocal microscopy analysis of LC3 double fluorescent cells. *P < 0.05; **P < 0.01; ***P < 0.001.** E-F.** Electron microscopy detected autophagic flow in SNU449 and HepG2 cells. **P < 0.01** G.** CCK-8 detected the sensitivity to doxorubicin in HCC cells after treatment with different conditions (transfected with miR-26b mimics, miR-26b mimic interference, and treated with rapamycin). *P < 0.05 vs Negative Control.

During autophagy, the level of LC3II protein expression serves as a read-out of the number of autophagosomes, and the conversion of LC3I to LC3II (LC3II/LC3I) is indicative of autophagic activity 33. The autophagy receptor, sequestosome 1 (SQSTM1, p62), connected the autophagic cargo to the autophagic membrane, and can be used as another widely marker of autophagic flux 34. The Western blot analysis showed that the combination of doxorubicin and miR-26b mimics could down-regulate the increased expression of USP9X, p53, and LC3II/LC3I following doxorubicin treatment, and up-regulated the decrease in p62 expression induced by doxorubicin **(Figure 4A).** The level of autophagy was determined by the total number of autophagosomes (mRFP + GFP) and autolysosomes (mRFP). As shown in **Figure 4B** and **C**, treatment with miR-26b mimic + doxorubicin could reduce the increase of doxorubicin-induced the total number of autophagosomes and autolysosomes in HepG2 and SNU449 cells **(Figure 4B-D)**. The characteristic autophagic ultrastructures were determined by TEM analysis. As shown in Figure 4E and F, compared with the control group, the red color indicated that the number of autophagosome was increased following doxorubicin treatment, whereas miR-26b over-expression could reduce the increase in the number of autophagosome induced by doxorubicin. Furthermore, the miR-26b mimic could inhibit autophagy activity. CCK-8 detected differences in the sensitivity between different groups (transfected with or without miR-26b mimics, and miR-26b mimics combined with rapamycin) to doxorubicin, showing that treatment with the miR-26b mimics enhanced the chemosensitivity of the HCC cells. This effect disappeared after the addition of rapamycin** (Figure 4G)**.

### Sp94dr/miR-26b mimic nanoparticles enhanced HCC cell doxorubicin sensitivity *in vivo*

**Figure 5:**
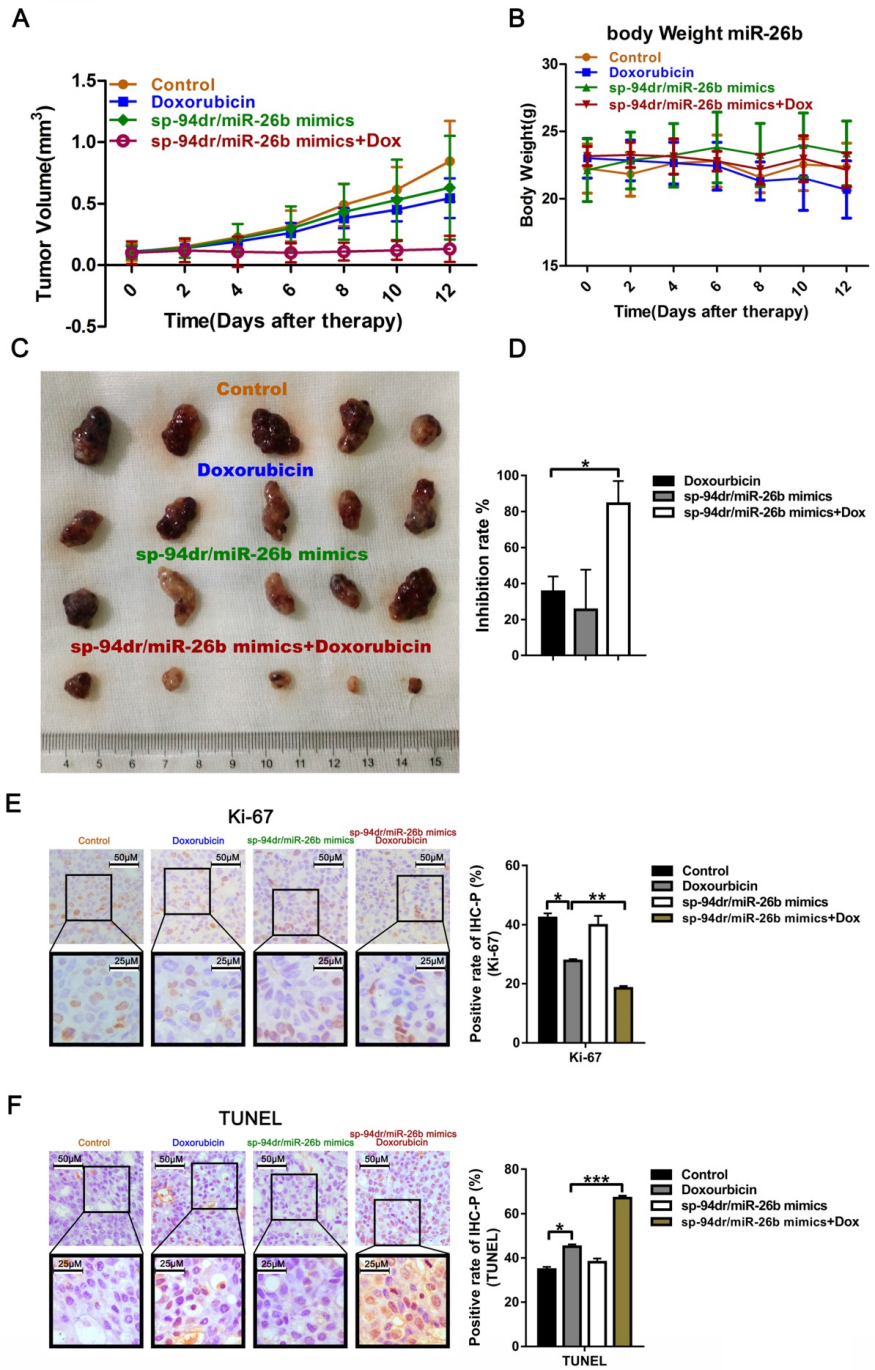
** Treatment with an miR-26b mimic inhibits tumor growth *in vivo*. A**. Growth curves of xenograft tumors treated with the control, doxorubicin, sp-94dr/miR-26b mimic, or doxorubicin plus with sp-94dr/miR-26b mimic (n = 6 per group). **B.** The mice were weighed on days 0-15 following treatment with either the control, doxorubicin, sp-94dr/miR-26b mimic, or doxorubicin plus with sp-94dr/miR-26b mimic. **C.** The morphology of the subcutaneous xenograft tumors in each of the different treatment groups. **D**. The tumor inhibition rate was determined following treatment with doxorubicin, sp-94dr/miR-26b mimic, or doxorubicin plus with sp-94dr/miR-26b mimic. *P < 0.05.** E.** Ki-67 staining was used to analyze the rate of Ki-67-positive staining in each of the treatment groups (40× magnification). *P < 0.05; **P < 0.01** F.** The presence of apoptotic cells was detected in each of the treatment groups by a TUNEL assay. *P < 0.05; ***P < 0.001**.**

We conducted further *in vivo* experiments using liver cancer-targeting peptides, sp94dr and miR-26b self-assembled nanoparticles to conduct experiments due to the cumbersome nature of drug administration *in vivo*. Treatment with the sp94dr/miR-26b mimic nanoparticles plus doxorubicin had an inhibitory effect on tumorigenesis *in vivo*, similar to the *in vitro* studies **(Figure 5A, C and D).** There was no significant change in the body weight of the mice after treatment in the different groups **(Figure 5B).** Ki-67 staining and a TUNEL assay found that treatment with the sp94dr/miR-26b mimic nanoparticles plus doxorubicin significantly decreased the tumor cell proliferation rates and increase tumor cell apoptosis compared to the other groups **(Figure 5E-F).**

**Figure 6:**
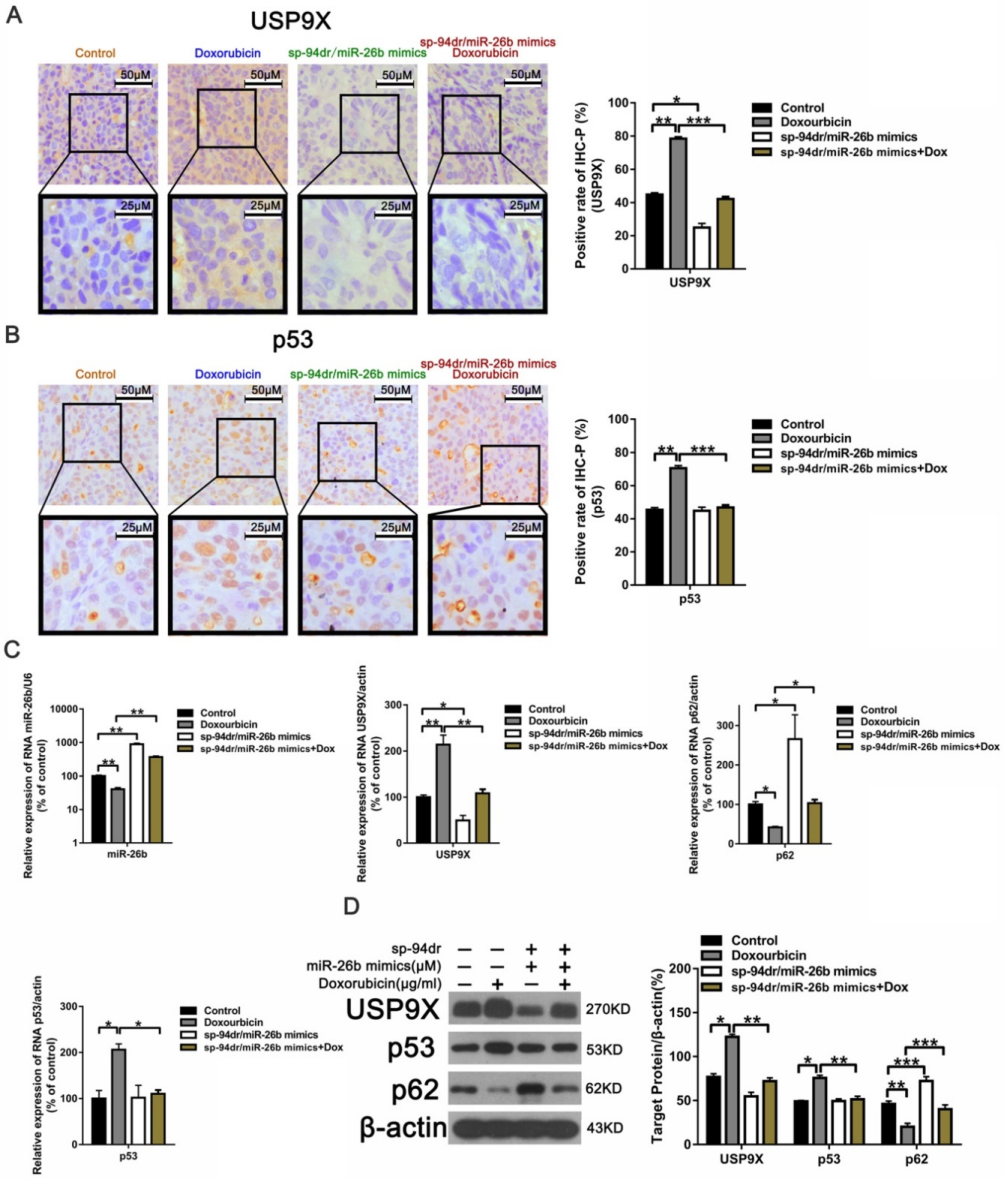
** Treatment with Sp-94dr/miR-26b mimic combined with doxorubicin down-regulates p53 and USP9X expression *in vivo*. A and B**. Immunohistochemistry-positive (IHC-P) cells were examined for the expression of USP9X and p53. *P < 0.05; **P < 0.01; ***P < 0.001. **C.** QRT-PCR analysis the expression of miR-26b, USP9X, and p53 in control, doxorubicin, sp-94dr/miR-26b mimic, and doxorubicin plus with sp-94dr/miR-26b mimic-treated cells. *P < 0.05; **P < 0.01; ***P < 0.001.** D.** USP9X, p53, and p62 protein expression were evaluated using a Western blot. *P < 0.05; **P < 0.01; ***P < 0.001.

**Figure 7:**
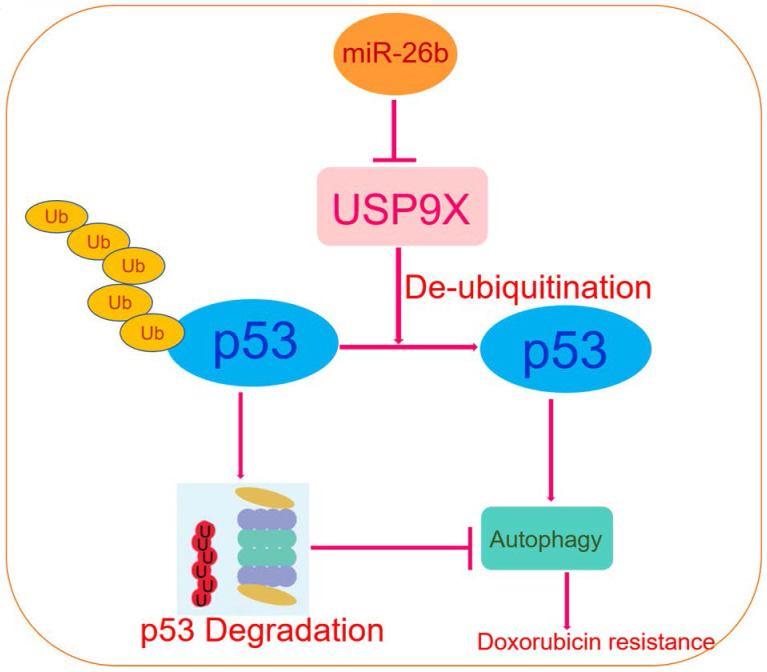
Schematic diagram of the regulatory mechanism of the miR-26b/USP9X/p53 axis in regulating HCC sensitivity to doxorubicin.

Our previously established chimeric peptide-condensed supramolecular nanoparticles were found to protect miR-26b from degradation 35. A schematic representation of the binding of miRNA and the polypeptide, SP-94dr, is presented in **Supplemental Figure 1 A.** The naked miR-26b band was significantly blocked when the SP-94dr/miR-26b ratio was 20:1 **(Supplemental Figure 1 B).** Agarose gel electrophoresis analysis showed that the RNase stability and miRNA serum stability were significantly increased after binding to SP-94dr **(Supplemental Figure 1 C and D).** Electron microscopy with the best ratio of nanoparticles (nanoparticles) was used to measure the self-assembled nanoparticles, and an average particle size of 135 nm ± 30 nm was recorded **(Supplemental Figure 1 E and F).** Furthermore, treatment with the SP-94dr/ miR-26b mimic nanoparticles plus doxorubicin was found to reduce the level of USP9X and p53 expression **(Figure 6A and B).** The level of miR-26b and p62 was upregulated following treatment with sp94dr/miR-26b mimic nanoparticles plus doxorubicin, whereas the expression of USP9X and p53 was downregulated compared with the control **(Figure 6C).** The level of USP9X and p53 protein expression following treated with doxorubicin was increased and p62 expression was decreased compared with the Control; however, the sp94dr/miR-26b mimic nanoparticles could reduce the up-regulation of USP9X and p53 induced by doxorubicin, increase doxorubicin induced the decrease level of p62** (Figure 6D).** Taken together, these results suggest that miR-26b increases doxorubicin sensitivity *in vivo*. Schematic diagram of the regulatory mechanism of the miR-26b/USP9X/p53 axis in regulating HCC sensitivity to doxorubicin was shown in Figure 7.

## Discussion

Numerous reports have demonstrated that miRNAs play a critical role in developing chemo-resistance based on their functions and targets 32, 33. miRNA-26b is encoded at 9p21.3, a vulnerable site in the genome, which has been reported to be missing in many HCC tumors and is downregulated in HCC cells 36. Moreover, the up-regulation of miR-26b can inhibit cellular proliferation and migration in HCC cells 37 and miR-26b can also suppress tumorigenicity and promote apoptosis in NSCLC cells by regulating Mcl-1 38. It has also been shown that miR-26b can improve the sensitivity of colorectal cancer cells to 5-FU *in vitro* and enhance the potency of 5-FU on the inhibition of tumor growth *in vivo* through downregulating P-glycoprotein (Pgp) protein expression 39. Therefore, determining important candidate miRNAs that regulate HCC chemotherapy resistance may help improve treatment. Our research focused on the role of miR-26b on doxorubicin chemoresistance, demonstrating that miR-26b expression was downregulated in HCC cells. Furthermore, treatment with the miR-26b mimic combined with doxorubicin could enhance the sensitivity to doxorubicin in all HCC cells, except in Hep3B cells. Moreover, our previous study found that WP1130 could enhance the sensitivity to doxorubicin in HCC cells; however, p53 was absent in Hep3B cells 22. As a tumor suppressor and transcription factor, p53 can regulate different cellular stress responses (e.g., activation of oncogene and DNA damage caused by genotoxic drug) and behaviors (e.g., apoptosis, cell cycle arrest, DNA repair, and cell growth) 40. The abnormal expression of p53 has been found in almost all types of cancers, and p53 mutations are related to a poor patient prognosis and resistance to chemotherapy 41, 42. Thus, we examined the expression of p53, and found that p53 expression was almost nonexistent in Hep3B cells. To determine the level of cell viability, p53 was knocked down after transfection with or without an miR-26b mimic plus doxorubicin in HCC cells, revealing that the role of the miR-26b mimic in doxorubicin sensitivity was ablated.

Autophagy is a process of maintaining homeostasis that is conserved in HCC cells following targeted therapy by promoting cell survival, and also induces resistance in a wide-range of cancer cells 29, 43. Indeed, inhibiting autophagy has been shown to overcome chemoresistance in many tumor cells 27, 44, 45 LC3‑I is present in the cytoplasm, while LC3‑II is localized inside and outside the membrane of autophagolysosomes 46. The degradation of LC3‑II in the autophagolysosome can be prevented by inhibiting autophagolysosome fusion. The number of LC3-II indicates the number of autophagosomes obtained during the conversion of LC3-I to LC3-II. Autophagy as strictly regulated by autophagy-related molecules and the number of LC3-II or LC3-II/LC3-I can reflect autophagy activity 47, 48. p62 is one of the most well-characterized autophagy receptors, and degradation via autophagy was found to be selected by linking cargo with autophagy mechanism 49. It has been reported that the up-regulation of miR-26b may enhance the sensitivity to doxorubicin and promote apoptosis via inhibiting autophagy in HCC cells 29. Furthermore, the main regulators of autophagy include the PI3K-Akt-mTOR pathway-associated molecules, RAS and p53 25. Several studies have shown that nuclear p53 stimulates cellular autophagy via the transactivation of multiple target genes, whereas cytoplasmic p53 inhibits autophagy in a transcription-independent manner. Therefore, the subcellular localization of p53 may determine the outcome of autophagy 50. Here, we investigated the role of autophagy in miR-26b-mediated regulation of doxorubicin resistance in HCC. Almost immediately, we examined LC3, double fluorescence and autophagic flow following transfection with an miR-26b mimic, and miR-26b mimic combined with doxorubicin. Interestingly, we found that the miR-26b mimic reduced the increased rate of LC3-II/LC3-I induced by doxorubicin and increased p62 expression, indicating that miR-26b upregulation suppressed autophagy. We subsequently found that following treatment with rapamycin combined with an miR-26b mimic, the increased doxorubicin sensitivity mediated by miR-26b had disappeared, suggesting that autophagy may be related to drug resistance in HCC cells.

Protein ubiquitination is regulated by various aspects of cellular physiology, including cell signaling and protein degradation, and it is also a reversible, post-translational modification 51. Moreover, DUBs can cleave ubiquitin from the substrate 52. In the human genome, approximately 100 encoded DUBs have been found, of which USP9X/FAM has been implicated in multiple physiological pathways 53. Accumulating evidence has revealed that USP9X is significantly up-regulated in human cancers, including pancreatic, breast, HCC, and lung cancers 54-57. Moreover, USP9X overexpression was found to be closely associated to apoptosis, metastasis, cellular proliferation, and chemotherapy resistance 54, 58. In this study, USP9X interference was shown to enhance HCC cell sensitivity to doxorubicin; however, the role of the miR-26b mimic disappeared. p53 has been determined to be the most common variant gene in cancers, and its regulation and stabilization of normal functions is essential. In addition, the ubiquitin-proteasome pathway (UPP) regulates p53 stability and is modulated by DUBs that can eliminate ubiquitin from p53. The ubiquitination of p53 can be reversed by DUBs, a group of enzymes that control the extent of p53 ubiquitination through deubiquitinating p53. Both ubiquitination or deubiquitylation regulate p53 inside the cells 59-61. MG132 acts as a proteasome inhibitor, further verifying that p53 downregulation was achieved by proteasome-dependent degradation via p53 ubiquitination. To further explore whether miR-26b mediates its synergistic cytotoxicity through p53 degradation, we treated cells with doxorubicin alone, or in combination with an miR-26b mimic or MG132 with doxorubicin and an miR-26b mimic. The results showed that MG132 reversed the inhibitory effect of miR-26b on p53 expression in the HCC cells following doxorubicin treatment. However, no significant differences in p53 expression were observed after treatment with the miR-26b mimic, suggesting that miR-26b promoted p53 degradation via USP9X. It has been reported that Tenovin-1 can protect p53 from degradation via mdm2, has little effect on p53 synthesis, and has also been identified as a p53 activator 22, 62. In addition, this effect was only found in wild type p53 but not in p53 mutant cells 63. Our study showed that Tenovin-1 protects p53 from ubiquitination-mediated degradation only in wild type p53 HepG2 cells. Tenovin-1 pretreatment enhances HCC cell resistance to doxorubicin following co-treatment with an miR-26b mimic, indicating that the USP9X/p53/ubiquitination/degradation pathway is important for miR-26b-mediated chemotherapy sensitivity. Furthermore, we established chimeric peptide-condensed supramolecular nanoparticles to protect miR-26b from degradation. Later, *in vivo* treatment with sp94dr/miR-26b mimic nanoparticles plus dox was associated with inhibitory effects on tumorigenesis. Furthermore, miR-26b was up-regulated, whereas USP9X and p53 expression were down-regulated, suggesting that miR-26b increases doxorubicin sensitivity via inhibiting USP9X and p53 *in vivo*.

## Conclusion

In conclusion, this study revealed that miR-26b regulation was a mechanism involved in the sensitivity of HCC cell lines to doxorubicin directly through USP9X-dependent degradation of p53 and regulation of autophagy (Figure 7). To our knowledge, this is the first report of the regulation of USP9X by miR-26b and its role in the chemotherapy response to doxorubicin. Furthermore, miR-26b may represent a potential gene-targeting approach for HCC treatment in the future.

## Supplementary Material

Supplementary figure S1.

## Figures and Tables

**Table 1:** IC_50_ values for doxorubicin in HCC cell lines with or without miR-26b mimic siRNA treatment.

HCC cell line	IC_50_ (μg/mL)^▲^	
	Doxorubicin	Doxorubicin + miR-26b mimic
SNU449	1.644±0.06229	0.6510±0.02349^ *^
Hep3B	0.7701±0.03647	0.7012±0.03443
HepG2	1.007±0.03633	0.5273±0.01735^*^
SNU387	0.7263±0.03554	0.2903±0.01201^*^

^▲^ IC_50_ concentrations of doxorubicin (μg/mL; mean [95% CI]). ^*^ *P* < 0.05 vs. Doxorubicin.
