# Supplementary material for: miR-26b enhances the sensitivity of hepatocellular carcinoma to Doxorubicin via USP9X-dependent degradation of p53 and regulation of autophagy
Source: Int J Biol Sci. 2021 Feb 8;17(3):781–95. doi: 10.7150/ijbs.52517 (PMC7975695; doi:10.7150/ijbs.52517)
Supplement: Supplementary file 1 — Supplementary figure S1. [file ijbsv17p0781s1.pdf]

Supplementary data

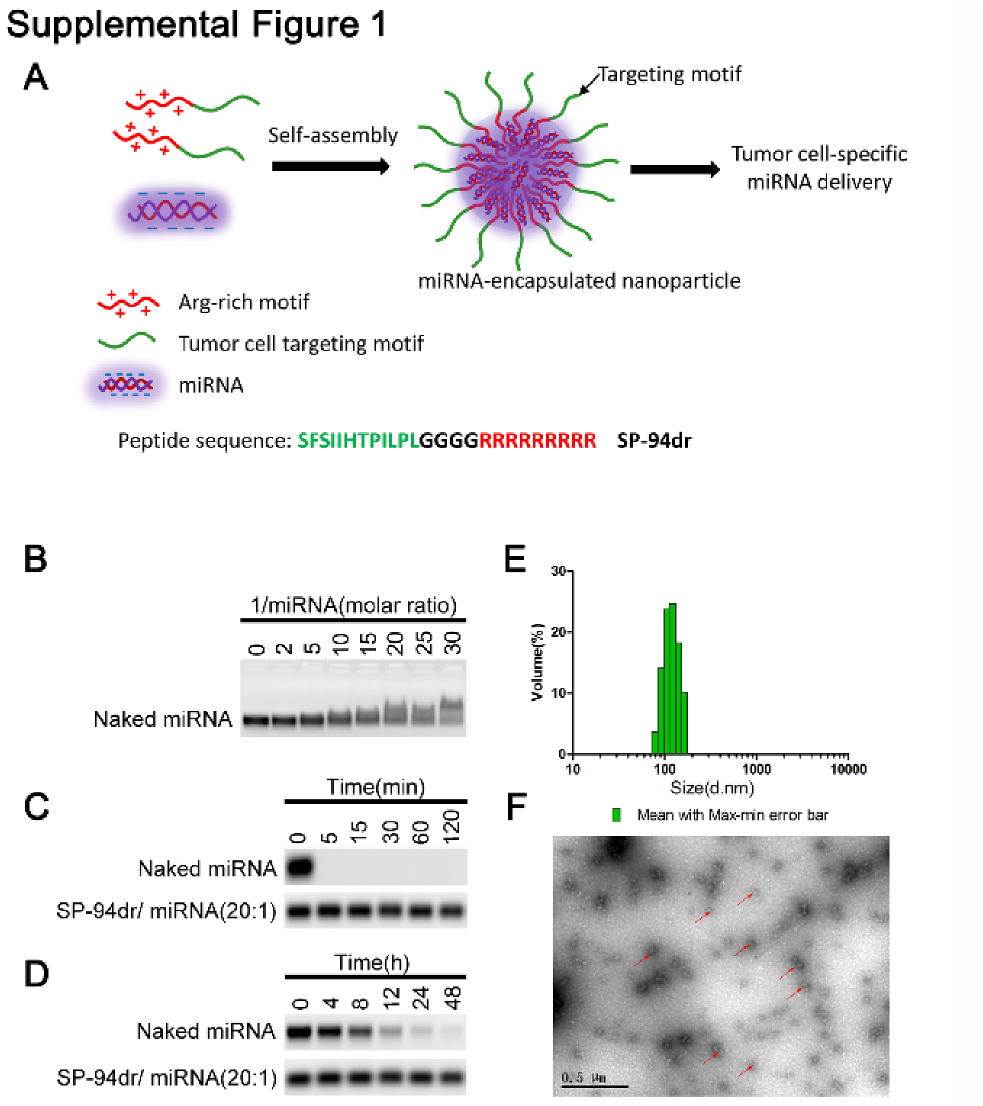

Supplementary Figure Legends

Supplemental Figure 1

A. Schematic representation of the binding of miRNA and polypeptides. **B.** An agarose electrophoresis assay determines the optimal ratio of polypeptides to miRNA. **C and D.** Agarose gel electrophoresis for RNase stability and serum stability. **E and F.** Electron microscopy with the best ratio of nanoparticles (nanoparticles) and particle size.
